# Closing cones create conical lamellae in secondary osteonal bone

**DOI:** 10.1098/rsos.220712

**Published:** 2022-08-10

**Authors:** Michael Doube

**Affiliations:** Department of Infectious Diseases and Public Health, City University of Hong Kong, Kowloon, Hong Kong

**Keywords:** bone, lamellae, secondary osteon, closing cone

## Abstract

Lamellae are sheets of mineralized collagen 1–20 µm thick, extending over hundreds of µm in bone tissue, occupying bone's structural hierarchy at a level above collagen fibres and osteocytes, and below osteons and trabeculae. Osteons are tubular arrangements of lamellae surrounding central neurovascular canals. Lamellae in osteons are usually described as concentric cylinders based on their annular appearance in transverse section. In this review, I provide a perspective on current understanding of the relationship between geometry of the bone formation front and the shape of lamellae produced at it, reaching the conclusion that the ‘closing cone’ bone formation front in secondary osteonal remodelling must necessarily result in cone-shaped lamellae in the mature secondary osteon. Secondary osteons replace primary osteons through a tunnelling process of bone turnover, meaning that conical lamellae may become more common in older and damaged bone which is at greatest risk of fracture. Visualization and measurement of three-dimensional lamellar shape over hundreds of microns is needed to provide data for accurate micromechanical simulations. Treating secondary osteonal lamellae as a ‘stack of cones’ rather than ‘nested cylinders’ may have important implications for our appreciation of bone's function as a load-bearing tissue and of its behaviour in fracture.

## 1. Introduction

Primary and secondary osteons are structural units within cortical (dense) bone, comprised of sheets of lamellar bone tissue that form rings around central neurovascular (Haversian) canals [1–3]. Despite their similar appearance in mature bone, the formation processes of primary and secondary osteons differ markedly. Primary osteons are formed upon the outer bone surface under the periosteum during bone growth [4], while secondary osteons are formed within cortical bone through a tunnelling process of bone removal and replacement [5–7]. Primary osteon formation occurs while the size and shape of the bone organ adapts to bring mechanical strains within a range of tolerance, referred to as ‘modelling’, whereas secondary osteon formation turns over bone tissue, repairing it and adjusting its microstructure, which is referred to as ‘remodelling’ [8].

In primary osteonal (and plexiform) bone formation, capillaries in the periosteum are surrounded by ridges of bone that extend peripherally forming walls that then join tangentially making a roof over the capillaries [4]. The walls, floor and roof of each tunnel are then filled in layer by layer by teams of osteoblasts that deposit unmineralized bone matrix (osteoid). A degree of synchrony is in play, leading to the completed osteons having annular rings of bony lamellae in transverse section. In longitudinal section, lamellae are usually depicted as stripes parallel to the osteon's axis, implying that primary osteonal lamellae are roughly cylindrical in three dimensions.

In the secondary osteon, old bone is removed by osteoclasts at a ‘cutting cone’ and osteoid is deposited by osteoblasts on a new forming surface, the ‘closing cone’ [5]. Contemporary cartoons [9–19] of the secondary osteonal cutting and closing cones show the resulting lamellar bone as cylindrical, as in primary osteons, concentric about the central Haversian canal, and usually intersecting rather than parallel to the sheet of osteoblasts on the forming surface. I contend that this familiar depiction of lamellae intersecting the cutting cone is incorrect and creates a misleading impression of the relationship between bone cells and the matrix that they form.

## 2. Osteoblasts, osteocytes, bone lining cells

Osteoblasts, osteocytes and bone lining cells are responsible for the production and maintenance of bone matrix [20], and derive from a perivascular [21] mesenchymal stem cell lineage [22], like fibroblasts [23], chondrocytes [24] and adipocytes [25]. Newly differentiated osteoblasts join a patch team at the leading edge of the closing cone where they become synchronized locally and systemically to produce a lamella by the deposition of aligned collagen fibrils [26]. The linear advancement of the secondary osteon of about 40 µm d^−1^ (in the dog, [27]) and the approximately 10 µm width of an osteoblast [28] implies that a new ring of osteoblasts differentiates at the leading edge, or base, of the closing cone about every 6 h. Osteoblasts can be relatively motile, taking a meandering path at 4–5 µm h^−1^ on flat surfaces such as calvaria while they seek substrate attachment, but are much less motile post-confluence [29]. Osteoid deposition pushes the osteoblast team toward the centre of the osteon in a direction orthogonal to the formation front. Osteoblast teams coordinate locally via gap junctions and hemichannels, mainly connexin43 [30], which transmit signals across the patch of osteoblasts and to nearby osteocytes [31]. Calcium ion concentration spikes appear spontaneously within individual osteoblasts and may propagate as an intercellular wave [32,33]. Signalling via the gap junction intercellular network is required for the orderly deposition of collagen by osteoblasts [34].

Three fates for osteoblasts are recognized: half or more undergo apoptosis [35], with the remainder burying themselves in osteoid and differentiating into long-lived osteocytes [36,37] or differentiating into senescent, flattened bone lining cells covering the Haversian canal surface. Bone lining cells may dedifferentiate back into osteoblasts [38,39]. Osteocytes liberated from their lacunae by bone resorption may be phagocytosed by multinucleated cells [40] or migrate out of their lacunae [41] and dedifferentiate into osteoblasts stimulated by the change in geometry of their surrounding matrix from bulk three-dimensional to a solid surface [42].

## 3. Osteoclasts

Osteoclasts are large, branching, bone resorbing cells that create the pits (Howship's lacunae) within which new bone matrix may be deposited [41]. In the secondary osteon, several osteoclasts work simultaneously and their resorption pits overlap to form a cutting cone [27], which proceeds longitudinally at about 40 µm d^−1^ and radially at about 7 µm day^−1^. Osteoclasts derive from haematopoietic tissue and are multinucleate as a result of fusion of precursor cells, in contrast to the mesenchymal lineage of osteoblasts. Recent evidence suggests that osteoclasts may be considered syncytia, with parts (osteomorphs) budding off osteoclasts where resorption is no longer needed, migrating, and fusing with osteoclasts where their activity is more in demand [43]. During remodelling, osteoclasts that contact osteoblasts and osteoid stop their resorbing activity and may recycle to the tip of the cutting cone [5,44]. Osteoclasts are in continuous communication with osteoblasts, osteocytes and bone lining cells via soluble and adsorbed signalling molecules [20].

In some situations, for example sudden reduction of habitual mechanical loading, resorption occurs in the osteonal space that is not immediately followed by bone apposition [45]. Upon resumption of high intensity loading the enlarged osteonal canals may be infilled [46], which if occurring simultaneously along the length of the cement line might lead to cylindrical lamellae. It is not currently known how common this phenomenon is, in contrast to the frequently observed cutting cone-closing cone dynamic, although irregular infilling was noted by Cohen & Harris in their maps of osteons with partial infilling and completed infilling [47].

## 4. Lamellae

**Figure 1.**
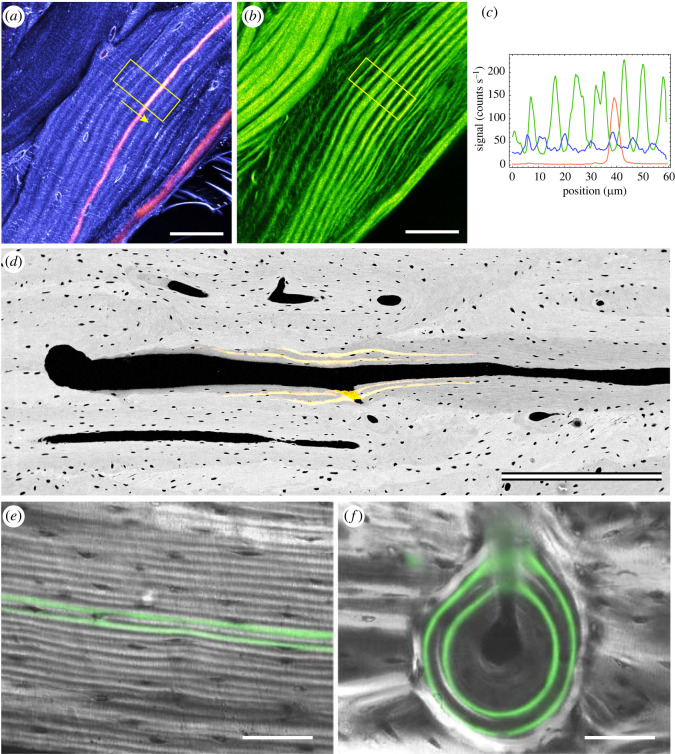
Fluorochrome labels identify lamellae; lamellae record the bone formation front's past activity. Third- (*a*) and second- (*b*) harmonic multiphoton microscopy identifies collagen orientation in lamellae (blue, green), while a calcein label (pink-red; *a*,*c*) perfectly maps to the lamellar contour. (*d*) Secondary osteon in longitudinal section progressing from right to left, imaged in backscattered electron scanning electron microscopy (BSE SEM; greyscale) and confocal scanning light microscopy (yellow). Note two calcein labels extending obliquely at 3–5° from the cement line and trailing off towards the Haversian canal. Close inspection of the BSE SEM image to the right of the calcein labels reveals lamellae that formed earlier in the closing cone's progression. (*e*,*f*) Combined transmitted circularly polarized light (greyscale) and confocal fluorescence microscopy (green) illustrate that intra-vital labels trace lamellae. Scale bars: (*a*,*b*,*e*,*f*) 50 µm; (*d*) 500 µm. Images reused under CC-BY terms from Genthial *et al*. [54] (*a–c*) and Bromage *et al*. [55] (*e,f*); (*d*) created de novo from archival data by Alan Boyde and may be reused under a CC-BY 4.0 licence.

Lamellae are a regular textural feature of bone matrix arising from periodic fluctuations in collagen production [48–50], with a concomitant long-range (multicellular) organization of collagen fibrils that align, then disorder, then realign in a new direction [2,50–53] (figure 1). An excellent recent review on sublamellar collagen organization and the main theoretical models may be found in Mitchell and van Heteren [50]. Lamellar bone is laid down in patches by teams of osteoblasts that work in coordination to produce osteoid with its collagen fibres roughly parallel throughout the patch [26]. Each patch is limited to ‘domains’ of a few hundred cells; collagen orientation in each lamella may result from several domains with different orientations [6,56]. Fibroblasts (of which osteoblasts are a specialized type) become aligned upon reaching confluence in a manner that relates to the edges bounding their patch [57]. Species-dependent lamellar periodicity may arise from centrally coordinated, synchronized, increases and decreases in osteoblast activity [55] with a diurnal frequency in small mammals that may be subject to occasional arrest [58], and a longer period in larger animals. Osteocytes start appearing about 9 days after osteoid deposition begins in the dog [59], which is enough time for one or two lamellae to form in larger mammalian species [60,61]. Osteocyte lacunae may appear in a regular relation to lamellae within the so-called ‘loose lamellae’ [48,62], but have also been reported to have no strict relation to lamellar type [63].

Bone-seeking fluorochrome labels given during life bind to bone matrix at the formation front through becoming incorporated with mineral that is accreting within freshly deposited osteoid [64]. Lead (Pb) salts have also been used to label bone formation [58]. The short period during which the label is present in circulation creates an activity timestamp: the label indicates that at a single moment bone deposition was occurring at the labelled sites simultaneously. Tetracyclines, calcein, xylenol and alizarin are commonly used for this purpose, with alizarin from madder root the eighteenth century precedent [65,66]. In all known cases the formation front labelled by fluorochromes or other markers corresponds directly to the disposition of the lamellae so produced [54,55,58] (figure 1).

Scant evidence exists on lamellar shape in three dimensions compared to the lower and higher hierarchical levels of bone tissue organization. Collagen orientation within lamellae, and osteons and trabeculae composed of lamellae, have been studied for decades [3,26,47,50,67], and in high detail in three dimensions [51,68]. It is crucial to appreciate that lamellae occupy a distinct position in bone's structural hierarchy [49], and are not simply regions of similar collagen orientation, or parts of higher-order structures such as osteons or trabeculae. In general, lamellar shape conforms to the three-dimensional shape of the sheet of osteoid deposited by osteoblast teams at the bone formation front [55]. Frasca *et al*. separated and imaged lamellae in scanning electron microscopy, however, they did not make a distinction between primary and secondary osteons, nor image sufficient length to determine long-range three-dimensional lamellar shape [69]. Mohsin *et al*. [70] cut 25 µm serial sections of ovine bone and imaged fluorochrome-labelled secondary osteons, but the thick sections and ‘wavy’ osteonal morphology limited detailed understanding of three-dimensional lamellar shape. Small- and wide-angle X-ray scattering (SAXS, WAXS) may be applied in three dimensions to image local collagen orientation [71–73]. Bone's mineralized collagen is highly scattering, making imaging of thick blocks of undecalcified material difficult in light microscopy. A combination of tissue clearing, with or without decalcification, intravital labels, second- or third-harmonic generation contrast [54,74,75] and optical [76] or physical [77,78] sectioning may be required to image lamellar geometry in three dimensions over the 100–1000 µm scale.

It is in little dispute that osteoblasts in secondary osteonal ‘basic multicellular units' are arranged in an approximately cone-shaped sheet, the closing cone. It follows that the lamellae produced at the closing cone should also be cone-shaped. Seminal work from the 1970s through to 1990 using fluorochrome-labelled bone deposition identified and illustrated staggered oblique bone formation fronts in longitudinally sectioned secondary osteons and used them to calculate the rate of osteoclastic resorption and dynamics of osteoblastic incorporation as osteocytes [27,59,79]. Another good example of an oblique formation front is figure 2 in Mohsin *et al*. [70]. The rates of axial resorption (c. 40 µm d^−1^) [27] and radial apposition (ca. 1–3 µm d^−1^) [46,80–82] imply a 1 : 40–3 : 40 gradient (1.4–4.3°) in the lamellae relative to the axis of the osteon. Figure 1*d* displays intra-vital calcein labels in a closing cone in cortical bone of a horse with an angular disposition of 3–7°. Although that may seem to be a small angle, it is similar to the slope of the sides of a typical bucket (2.5–6°) and a little less than ‘witch's hat’ traffic cones (8.5°), which are designed for ready stacking. Lamellar thickness tapers off at the apex of the closing cone, due to reduced width of the loose lamellae as a function of proximity to the Haversian canal [62]. There is no ‘cut edge’ of lamellae deep to bone lining cells, instead completed osteons are lined by a continuous layer of branching collagen fibrils [83]. The tapering off and sealing of the boundary may be associated with decreasing osteoid production from osteoblasts as they differentiate into bone lining cells or apoptose at the completion of osteonal infilling.

**Figure 2.**
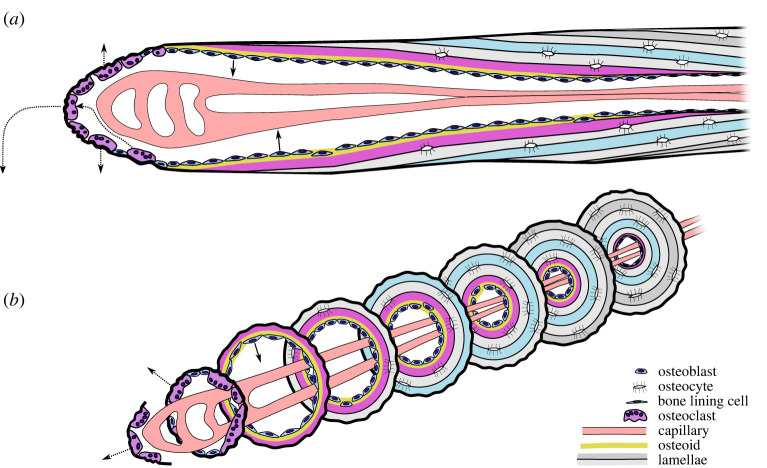
Updated schematic illustrating the relationship between cells and lamellae in the secondary osteon in longitudinal (*a*) and transverse (*b*) sections. Osteoclasts (movements in dashed arrows) resorb axially and radially, followed by a sinusoidal capillary bud. Osteoclasts recycle within the cutting cone, detaching from the cutting cone once osteoid production and osteoblast density reach critical thresholds. The cutting cone advances by the resorption of old bone. The closing cone advances by the recruitment of newly differentiated osteoblasts to its leading edge. New osteoblast recruits become synchronized to the closing cone osteoblast team and to systemic regulatory factors, to produce a roughly conical layer of osteoid. Periodic fluctuations in osteoid production and osteoblast orientation result in lamellae that follow the contour of the osteoid seam. Note the pennate arrangement of lamellae in longitudinal section, here swept at 4–5°, which in three dimensions may be understood as a stack of cones. Two lamellae are indicated in magenta and blue to aid the identification of this conical geometry. Lamellar spacing exaggerated about 3× for clarity. Some osteoblasts are buried in osteoid and differentiate into osteocytes. Old osteoblasts that have completed osteoid deposition but which did not become osteocytes may be retained as very thin bone lining cells or undergo apoptosis (not shown). Osteoblasts, bone lining cells and osteocytes in each transverse plane belong to the same generation and are about the same age, with the youngest to the left, and oldest to the right, of this figure. Figure may be reused and modified under a CC-BY 4.0 licence.

## 5. Origin of the nested cylinder lamellar model

**Figure 3.**
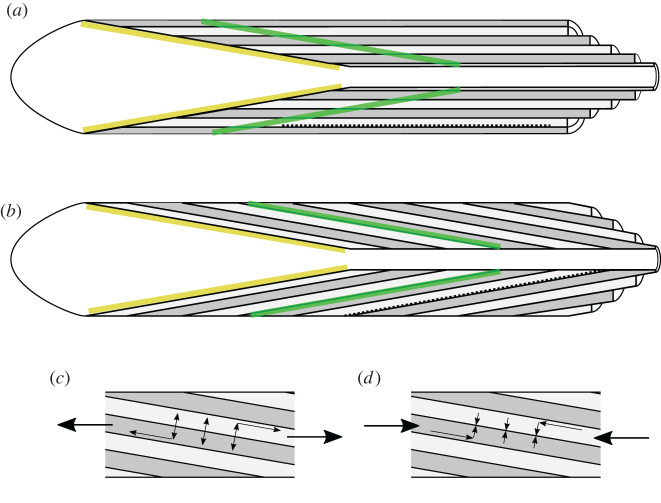
Schematic drawings illustrating (*a*) incorrect and (*b*) corrected lamellar orientation with respect to the secondary osteonal formation front at the closing cone (yellow), and an intra-vital label (green). Cutting cone to the left, osteon cut-away to the right; cells and vessels omitted for clarity. Note the lamellae intersecting the formation front and intravital label in (*a*) and parallel to them in (*b*). Cylindrical lamellae in (*a*) may shear past each other under tension and compression but cone-shaped lamellae in (*b*) might tend to jam or slip; this hypothetical model is illustrated in (*c*) tension and (*d*) compression acting on conical lamellae. The degree of tension or compression acting across the interlamellar boundary would relate to the angle of the applied force (large arrows) relative to lamellar orientation. Lamellae in (*a*) may direct crack growth (dashed line) parallel to the Haversian canal, whereas in (*b*) lamellae may allow crack growth from cement line to Haversian canal.

The idea that the lamellae of secondary osteons are cylindrical is now so pervasive as to be referred to in passing as accepted knowledge (e.g. [51], citing [10,84]). Enlow described cylindrical lamellae in secondary osteons in 1969 [85], but did not observe cutting cone – closing cone geometry perhaps due to a preponderance of transverse sections in his work [86], in which the longitudinal progression of secondary osteonal remodelling is not obvious. An early, and perhaps the first, depiction of cylindrical lamellae was in Parfitt [14], which was reproduced alongside some attractive isometric projections of a hemi-osteon in a 1994 review [11]. Robling and Stout continued the cylindrical lamellar trend in their 1999 work on drifting osteons [18], and Currey amplified it in his influential *Bones: Structure and Mechanics* [10]. In the dental field, Roberts *et al*. have propagated the cylindrical lamellar model while illustrating in the same drawing oblique fluorochrome labels cutting across the lamellae [87,88]. In an attempt to reconcile the apparent conflict between cylindrical lamellae and oblique formation front, Martin and Burr [84] proposed a concept by which the osteoblasts each work on the step-like leading edges of unfinished lamellae, like spectators seated on the ring-shaped benches of a Roman arena, which later appeared in a model by Currey [89]. The arena model would imply a discontinuity of incomplete lamellar edges that has never been observed in real samples, but is tacitly accepted by the proliferation of illustrations showing closing cones intersecting cylindrical lamellae. In all published data where both lamellae and fluorochrome labels appear, the labels are parallel to and delineate lamellae, which rules out the Roman arena seating model of Martin & Burr [84] that would imply labels cutting obliquely across lamellae as illustrated by Roberts *et al*. [87] (figure 3).

## 6. Implications of the stack of cones lamellar model

Recognition of the lamellar organization within bone has several important corollaries that relate to our understanding of bone's mechanical behaviour. Fracture [90], fatigue microdamage [91–93], osteonal pullout [94–96] and pushout [97] occur at interlamellar boundaries. Oblique lamellae might deflect crack growth toward the Haversian canal or cement line rather than along the osteon's length (figure 3*a,b*). Oblique fracture propagation appears to occur in single osteon bending tests [98]. Models that assume osteons are composed of concentric cylinders [99–105] that may find interlamellar shearing behaviour under tension, compression and torsion as the surfaces are all parallel or orthogonal to the loading axis. On the other hand, a stack of cones could behave as a series of wedges under compression, converting an axial force into radial compression as a function of wedge angle. Under tension, the wedge angle might create a tendency for lamellar separation to occur (figure 3*c,d*).

As bone gets older, more of it has been replaced by remodelling, potentially several times in a decades-long lifetime [106], depending on the specific skeletal element and species [107]. Secondary osteons may occur more frequently in regions where strains are higher as they have been recruited to repair microdamage [45,108–111]. Thus, the cylindrical lamellae model becomes increasingly inaccurate when discussing the highly loaded bone of athletes and military recruits, and the older bone of aged individuals, who are most at risk of stress or fragility fracture. In small species' bones, circumferential lamellae surrounding the medullary cavity may adopt a similar conical geometry (Enlow's ‘V’ principle), albeit over a larger spatial extent at millimetre scale rather than the few 100 µm typical of osteons.

Obliquity of lamellae relative to the optical axis may lead to bias in measurements of bright and dark lamellae under polarized light microscopy, which may occur even at angles smaller than 2° [63] that are, according to the stack of cones model, the usual case in secondary osteons. Thus, interpretations relating to collagen orientation may become confounded by the proportion of bone that has been turned over by secondary remodelling.

Idealizing osteons as precise nested cylinders or stacks of cones departs substantially from the reality that osteons form an overlapping, anastomosing network with multiple branches, buds, tapering blind ends and incomplete infilling [47,68,112,113]. Rather than being circular in transverse section, osteons are often elliptical [114,115], with crescent moon lamellae [61,116] or drifting morphology [18]. Secondary osteons are uncommon in many species [117] and are absent from those with a body mass less than about 2 kg. This does not preclude the need to recognize that lamellar shape follows that of the forming surface as it moves through the organ, which in an idealized secondary osteon closing cone is rather more cone-like than cylinder-like.

## 7. Conclusion

Despite its basic nature, the conical disposition of secondary osteonal lamellae appears up to now to have been omitted from discussions of bone remodelling and biomechanics, perhaps due to the fundamental relation between the bone formation front and lamellar shape in three dimensions rarely, if ever, being explicitly stated. The depiction of cylindrical lamellae in secondary osteons might result from a conflation of two distinct processes, primary osteonal bone formation, in which the bone formation front may be roughly cylindrical, and secondary bone formation, in which the bone formation front may be roughly conical. Confusion between primary and secondary osteonal bone formation geometry may disrupt a ready understanding of osteoblast and osteocyte movement relative to the cutting and closing cones and the relation between cells and lamellae in secondary osteons. Correct depiction of the arrangement of the main components of the secondary osteon is essential to dispel confusion about the relations between cells and matrix in this dynamic, moving process in bone. Adoption of the ‘stack of cones’ model proposed here, showing lamellae parallel to the bone formation front's closing cone, may aid fundamental understanding of secondary osteonal remodelling dynamics and the mechanical behaviour of mature cortical bone.

## Data Availability

Editable versions of figure 2 are available at doi:10.6084/m9.figshare.19954163.v2
